# Structural analysis of the regulatory mechanism of MarR protein Rv2887 in *M. tuberculosis*

**DOI:** 10.1038/s41598-017-01705-4

**Published:** 2017-07-25

**Authors:** Yun-Rong Gao, De-Feng Li, Joy Fleming, Ya-Feng Zhou, Ying Liu, Jiao-Yu Deng, Lin Zhou, Jie Zhou, Guo-Feng Zhu, Xian-En Zhang, Da-Cheng Wang, Li-Jun Bi

**Affiliations:** 1 School of Stomatology and Medicine, Foshan University, Foshan, 528000 Guangdong Province China; 2 Key Laboratory of RNA Biology & National Laboratory of Biomacromolecules, Institute of Biophysics, Chinese Academy of Sciences, Beijing, 100101 China; 3 Shanghai Key Laboratory of New Drug Design, School of Pharmacy, East China University of Science and Technology, Shanghai, 200237 China; 4 State Key Laboratory of Virology, Wuhan Institute of Virology, Chinese Academy of Sciences, Wuhan, 430071 China; 5 Center for Tuberculosis Control of Guangdong Province, Guangzhou, 510630 China; 6 The 4th Peoples’ Hospital, Foshan, 528000 Guangdong Province China; 7 Guangdong Province Key Laboratory of TB Systems Biology and Translational Medicine, Foshan, 528000 China; 8 University of the Chinese Academy of Sciences, Beijing, 100049 China

## Abstract

MarR family proteins are transcriptional regulators that control expression of bacterial proteins involved in metabolism, virulence, stress responses and multi-drug resistance, mainly via ligand-mediated attenuation of DNA binding. Greater understanding of their underlying regulatory mechanism may open up new avenues for the effective treatment of bacterial infections. To gain molecular insight into the mechanism of Rv2887, a MarR family protein in *M. tuberculosis*, we first showed that it binds salicylate (SA) and para-aminosalicylic acid (PAS), its structural analogue and an antitubercular drug, in a 1:1 stoichiometry with high affinity. Subsequent determination and analysis of Rv2887 crystal structures in *apo* form, and in complex with SA, PAS and DNA showed that SA and PAS bind to Rv2887 at similar sites, and that Rv2887 interacts with DNA mainly by insertion of helix α4 into the major groove. Ligand binding triggers rotation of the wHTH domain of Rv2887 toward the dimerization domain, causing changes in protein conformation such that it can no longer bind to a 27 bp recognition sequence in the upstream region of gene Rv0560c. The structures provided here lay a foundation for the design of small molecules that target Rv2887, a potential new approach for the development of anti-mycobacterials.

## Introduction

MarR family proteins are transcriptional factors which regulate the expression of genes involved in a wide variety of cellular processes, including stress responses, virulence, metabolic pathways and antibiotic resistance^1–4^, mainly via ligand-mediated attenuation of DNA binding. Given the importance of this family of proteins in antibiotic resistance, virulence and catabolism, greater understanding of the mechanism of their regulation may open up new avenues for the effective treatment of bacterial infections^3, 4^.

MarR proteins were first identified in the multidrug resistant *E. coli* strain K-12^5–7^ where they were associated with a mild multiple antibiotic resistant (‘mar’) phenotype that is induced by exogenous salicylic acid (SA). MarR homologues are found in many archaea and bacteria^8–12^, including *Mycobacterium tuberculosis*, the etiological agent of tuberculosis (TB), an ancient disease that caused an estimated 1.8 million deaths in 2015^13^ and is becoming an increasing public health concern due to the emergence and significant spread of drug resistant strains.

MarR family DNA binding proteins are typically repressors and are characterized by their triangular tertiary structure and the presence of a wHTH DNA-binding domain^14–16^. They generally form homodimers to bind to dsDNA, and this interaction is regulated by specific small molecule ligands, such as salicylate (SA), ethidium, and benzoate^2, 3, 17^. Ligand binding typically causes the wHTH motif to rotate upward toward its dimerization interface in such a way that proteins are no longer able to bind to DNA^1, 3, 4^. In some cases, DNA binding can be modulated by the oxidation of cysteine residues in the MarR protein.

The biological roles and mechanisms of the eight genes in the genome of *M. tuberculosis* annotated as MarR-like proteins have only recently received attention. While MarR-like regulator Rv1404 is reported to coordinate adaptation to acid stress by regulating the expression of Rv1405c, a virulence-associated methyltransferase^18^, Rv0678 controls transcription of the MmpS5-MmpL5 transporter^19^. Rv0880 and Rv2887 have recently been associated with drug resistance^20–22^, Rv0880 being shown to be involved along with transcriptional regulator Rv0324 in tolerance to the drug bedaquiline^21^, and Rv2887 to be important for sensitivity to a promising new imidazopyridine-based drug candidate MP-III-71 and to pyrido-benzimidazole ‘14’^20, 22^. Involvement of Rv0560c, a SAM-dependent methyltransferase, was implicated in the mechanism of action of Rv2887 on MP-III-71^22^, and Rv0560c was shown to *N*-methylate 14, abolishing its mycobactericidal activity^20^, but the structural and mechanistic basis of the regulatory action of Rv2887 on Rv0560c has yet to be fully elucidated. As SA, a common ligand of MarR family proteins, and structurally-related compounds, including the second-line anti-tubercular drug para-aminosalicylic acid (PAS), also induce high levels of Rv0560c expression^23^, and reports indicate that Rv2887 has a binding site in the Rv0558-Rv0560c gene cluster^24, 25^, we set out to investigate if binding of SA and structurally-related compounds PAS and gemfibrozil to MarR-like protein Rv2887 in *M. tuberculosis* results in the expression of Rv0560c, and to determine the structural basis of this action.

Using molecular-genetic, biochemical, biophysical and structural analyses, we provide detailed molecular insight into the regulatory mechanism of MarR family protein Rv2887 in *M. tuberculosis*. We show that Rv2887 binds ligands SA, PAS and gemfibrozil, and determine its precise DNA binding sequence in the promoter of Rv0560c. In addition, by comparing structures of *apo*-, ligand-bound and DNA-bound Rv2887 obtained by X-ray crystallography, we elucidate its mechanism of DNA recognition. The structures described herein open new pathways for the design of small molecules that target Rv2887, a potential new approach for the development of anti-mycobacterials.

## Results

### Protein Rv2887 binds to a sequence upstream of the Rv0560c gene

**Figure 1:**
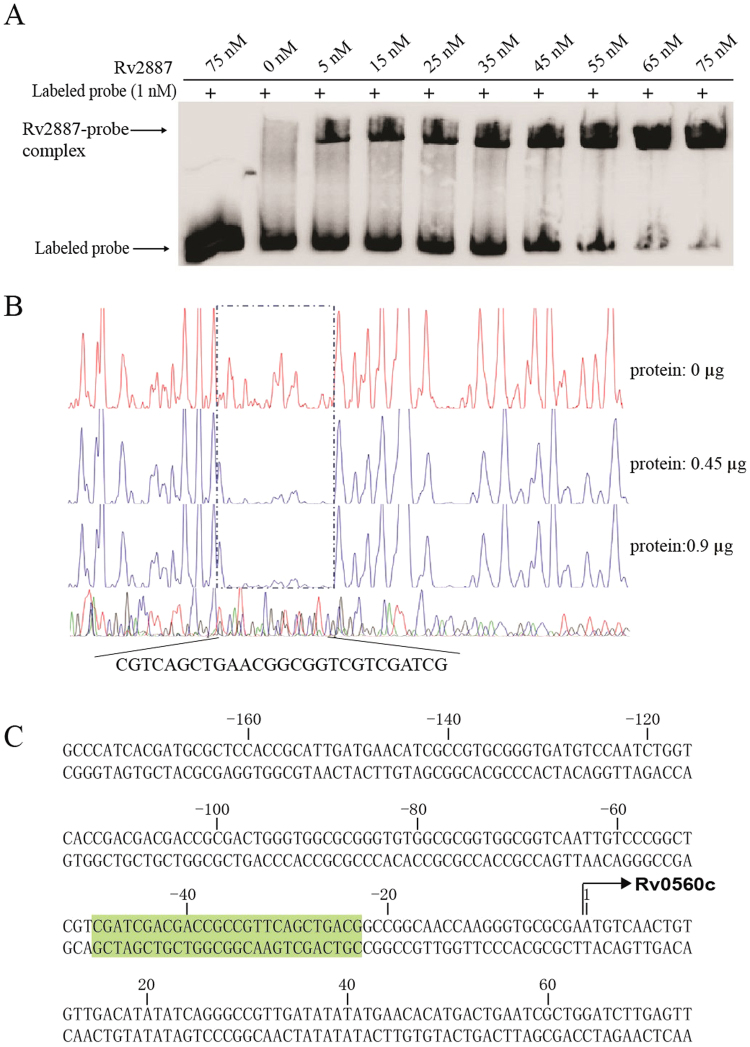
MarR family protein Rv2887 binds to a sequence upstream of the Rv0560c gene. (**A**) EMSA experiment using a PCR-amplified DNA probe spanning the upstream region of Rv0560c. Migration of the DNA probe was retarded compared to free-labeled DNA on addition of Rv2887. A labeled random DNA sequence of the same length as the target probe was used as a control (lane 1) (**B**) Dye primer-based DNase I footprinting shows that Rv2887 binds directly to a sequence upstream of Rv0560c. Electropherograms indicating the protection pattern of the region upstream of Rv0560c on digestion with Dnase I after incubation with (I) 0 μg (II) 0.45 μg or (III) 0.9 μg Rv2887 protein. (**C**) The protected DNA sequence. The DNA sequence upstream of Rv0560c showing the Rv2887 binding site (highlighted in light green).

To provide direct evidence that MarR-like protein Rv2887 regulates Rv0560c transcription, we performed EMSA using a DNA probe containing the sequence −175 to +75 bp upstream of the start site of the Rv0560c transcript. Migration of the probe was clearly retarded upon addition of purified Rv2887, and probe DNA shifted in a concentration-dependent manner, indicating that Rv2887 binds to the upstream sequence of Rv0560c *in vitro* (Fig. 1A), consistent with a recent report by Warrier *et al*.^20^.

To further define the binding site of Rv2887 in the upstream region of Rv0560c, we performed DNase I footprinting. Comparing electropherograms from various concentrations of dimeric Rv2887 protein identified a specific 27 bp DNA sequence, CGTCAGCTGAACGGCGGTCGTCGATCG, which is protected by Rv2887 protein (Fig. 1B). The bound region, positions −49 to −23 (Fig. 1C), is a GC-rich sequence that contains a potential palindromic motif, CGATCG, a typical characteristic of binding sequences of MarR family proteins^1, 3, 4^. This sequence is upstream of the annotated translational start site of Rv0560c and does not overlap with a region in a sequence downstream of the annotated translational start site suggested as a potential repressor binding site by Schleusser and Parish based on changes in promoter strength when mutations were introduced into this region^23^, suggesting the possibility that Rv0560c is regulated by more than one repressor.

### SA and structurally-related compounds bind to MarR protein Rv2887

**Figure 2:**
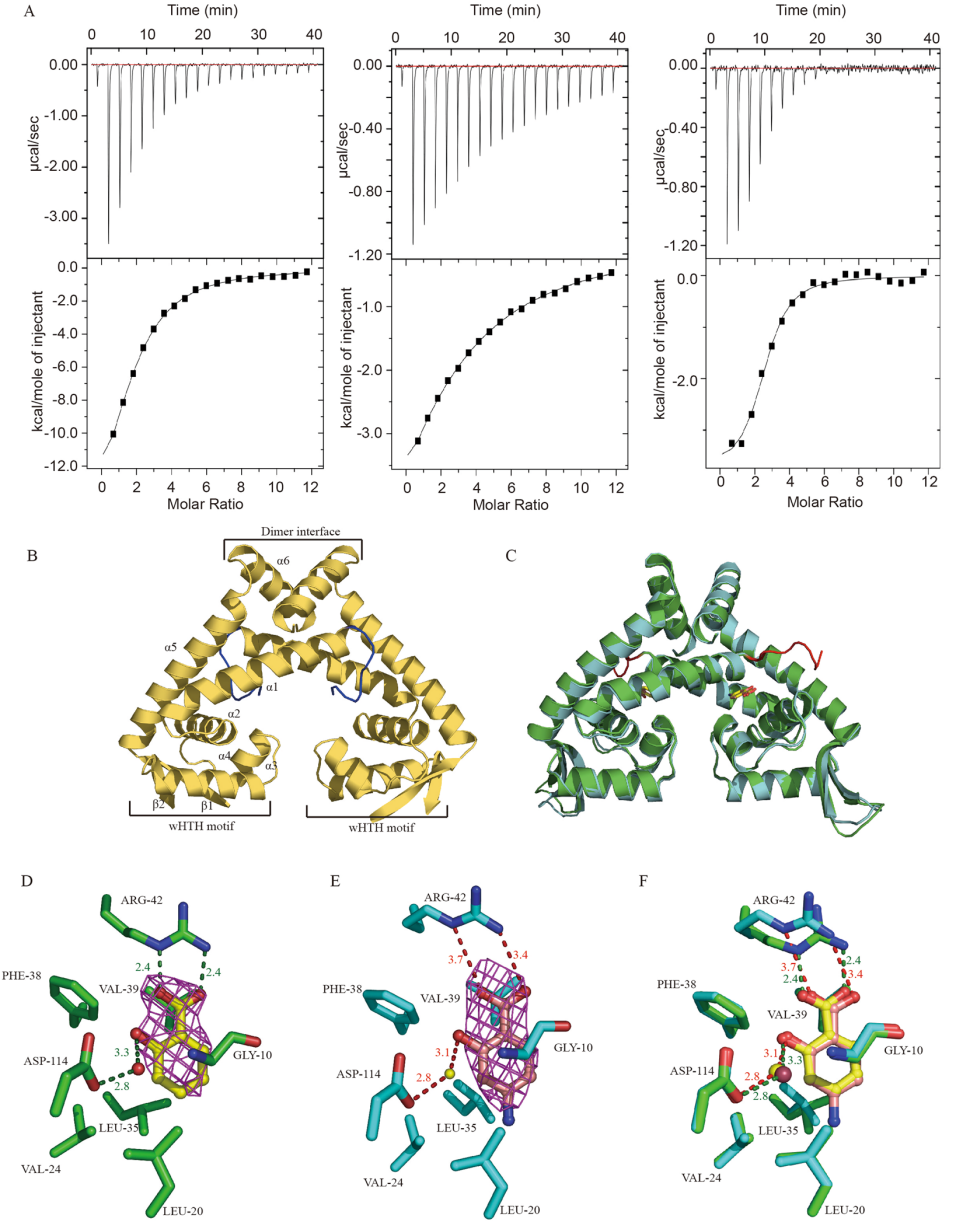
Rv2887-SA and Rv2887-PAS complex structures show that SA and PAS bind to Rv2887 in a similar manner (**A**) Representative binding isotherms of SA (left) PAS (centre) and gemfibrozil (right) titrated into Rv2887, as determined by isothermal titration calorimetry. (**B**) Secondary structure of apo Rv2887. Elements of one of the subunits, the dimerization interface and the winged helix-turn-helix motif (wHTH) are labelled. (**C**) Secondary structure superposition of the Rv2887-PAS dimer (blue) and the Rv2887-SA dimer (green). (**D**) Stereo image of the SA binding pocket; residues from Rv2887 are shown as sticks. Polar contacts are shown as green dashed lines, and the red sphere is a water molecule. (**E**) Stereo image of the PAS binding pocket; residues from Rv2887 are shown as sticks. Ligands SA and PAS are surrounded by ligand-omit 1Fo-Fc electron density maps (magenta) contoured and 3.0 σ. Polar contacts are shown as red dashed lines, and the yellow sphere is a water molecule. (**F**) Stereo image of the superposition of the PAS binding pocket and the SA binding pocket.

MarR homologues are known to bind a variety of ligand compounds, including SA and ethidium^2, 3, 17^, and, as mentioned above, Rv0560c has previously been shown to be strongly induced by SA, PAS, gemfibrozil and other structurally-related compounds^23^. Here, to determine whether SA, PAS and gemfibrozil are ligands of MarR protein Rv2887, and to see if they induce the expression of Rv0560c via MarR-like protein Rv2887 in a similar manner, we first used isothermal titration calorimetry to investigate the binding of SA and its structural analogue PAS to Rv2887. We found that SA binds tightly to Rv2887 with a binding affinity constant, Ka, of 2.45E4 ± 1.23E3 M^−1^, PAS with a Ka of 5.47E3 ± 307 M^−1^, and gemfibrozil with a Ka of 1.26E5 ± 2.11E4 M^−1^ (Fig. 2A). The molecular ratio in these ligand binding reactions was two ligands per Rv2887 dimer.

### Crystal structures of the Rv2887-SA and Rv2887-PAS complexes show that SA and PAS bind to Rv2887 in a similar manner

To elucidate the molecular mechanism of the MarR family protein Rv2887 in more detail, we first determined the crystal structure of *apo*-Rv2887 (Supplementary Table S1). The global structure of Rv2887 resembles the structures of other MarR family proteins^9, 14, 19, 26–28^. Rv2887 is present as a dimer with a triangular shape and belongs to the α/β family of proteins. It consists of six α-helices and two β-strands, arranged in the order α1-α2-α3-α4-β1-β2-α5-α6 in the primary structure (Fig. 2B). Each subunit is composed of two functional domains: helices α1, α5, and α6 form the dimerization domain, and helices α3 and α4 form a helix-turn-helix (HTH) DNA-binding domain. A wing motif comprising two antiparallel β-strands and their connecting loop is present between helices α4 and α5. This loop was disordered in the *apo* crystal form and could not be built.

To gain insight into the location and manner by which ligands SA, PAS and gemfibrozil bind to Rv2887, we set out to determine the crystal structures of Rv2887-ligand complexes (Supplementary Table S1), obtaining crystals of sufficient quality for structural resolution for the Rv2887-SA and Rv2887-PAS complexes, but not for the Rv2887-gemfibrozil complex. Further investigation of gemfibrozil as a ligand was thus reserved for a subsequent study. The crystal structures of these complexes indicate that one Rv2887 protomer binds one ligand molecule (Fig. 2C), consistent with our findings from ITC studies that SA and PAS bind to Rv2887 in a 1:1 ratio. Two molecules of SA/PAS bind Rv2887 in two deep symmetry-related pockets at the dimerization interface, where each ligand interacts with residues from both protomers. The protein conformations in the Rv2887-SA and Rv2887-PAS complex structures are identical, with Cα atoms having an r.m.s.d. of 0.42 Å (Fig. 2C). Rv2887 binds both SA and PAS in the same cavity surrounded by helices α1, α2, α3, and α5 from one protomer and helix α1 from the other protomer. Residue Arg42 of Rv2887 forms two hydrogen bonds with the carboxylic acid groups of SA and PAS, and residue Asp114 interacts with their hydroxyl groups via a water molecule bridge (Fig. 2D,E). The hydrogen bond distances between residue Arg42 and the carbohydrate group of the ligand in the Rv2887-SA complex are shorter than those in the Rv2887-PAS complex (Fig. 2F), possibly explaining the higher affinity with which Rv2887 binds SA compared to PAS, as described above. Residues Leu20, Val24, Leu35, Phe38 and Val39 of one protomer interact with the phenol group of SA/PAS by van der Waals interactions, and residue Gly10 of the other protomer stacks to the phenol group plane (Fig. 2D–F). To determine the functional importance of Arg42 and Asp114 in ligand binding, we used site-directed mutagenesis of Rv2887 to generate a protein in which these residues were substituted with an alanine (R42A and D114A), then examined ligand binding in these mutant proteins using ITC. These substitutions attenuated the ability of Rv2887 to bind SA (Supplementary Table S2), indicating that Arg42 and Asp114 are very important for binding SA. Alignment of the amino acid sequences of Rv2887 with other *M. tuberculosis* SA-binding MarR family proteins and those from other species (Supplementary Fig. S1) indicates that SA/PAS binding-related residues are not conserved among MarR family proteins. SA-binding itself varies widely among MarR family proteins; the number of SA ligands and the degree of conformational change that occurs on ligand binding vary between proteins, for example, while *S. epidermidis* TcaR binds eight SA molecules, the *E. coli* MarR structure has two SA molecules per dimer, and while *M. thermoautotrophicum* MTH313 undergoes large asymmetrical conformational changes on SA binding, SA-binding causes no conformational change in *S. tokodaii* ST1710. In summary, *M. tuberculosis* Rv2887 binds SA and PAS in a similar manner without causing any significant conformational change to local residues.

### SA/PAS binding attenuates Rv2887-DNA binding

**Figure 3:**
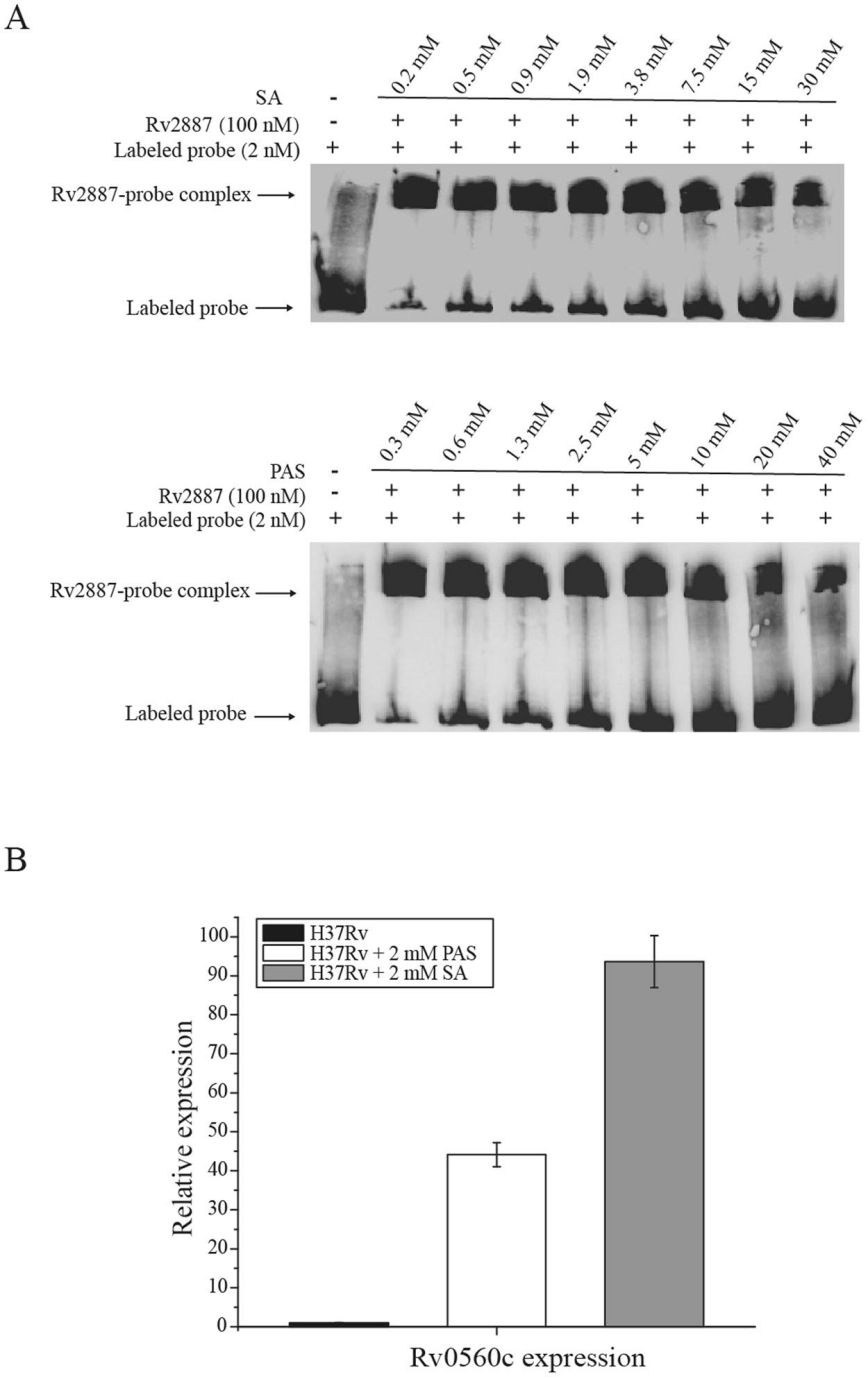
SA/PAS binding attenuates Rv2887 DNA-binding (**A**) EMSA of the Rv2887-DNA complex in the presence of increasing amounts of SA (upper panel) and PAS (lower panel). Dissociation of DNA from Rv2887 was observed on addition of increasing amounts of SA or PAS. (**B**) qRT-PCR analysis of Rv0560c expression in *M. tuberculosis* H37Rv. Expression of Rv0560c was measured in the presence and absence of 2 mM PAS or 2 mM SA. Values normalized to *sigA* are expressed relative to the level of Rv0560c in the absence of PAS and SA. Error bars indicate the SD.

MarR family proteins typically regulate transcription through ligand-mediated attenuation of DNA binding. Having shown that Rv2887 binds upstream of Rv0560c, and ligands SA and PAS interact with Rv2887, we then investigated if interactions with these ligands affect the DNA binding ability of Rv2887. EMSA assays using purified Rv2887 protein and a DNA fragment containing the upstream region of Rv0560c in the presence of SA and PAS showed that addition of SA or PAS to the Rv2887-DNA complex resulted in loss of the retarded band, indicating separation of the protein and DNA components (Fig. 3A). These observations suggest that binding of SA/PAS promotes the dissociation of Rv2887 from the upstream region of Rv0560c *in vitro*.

We corroborated these *in vitro* observations by RT-PCR analyses; Rv0560c transcripts increased about 90-fold and 40-fold, respectively, after *M. tuberculosis* cultures were incubated for 90 min in media supplemented with 2 mM of SA or PAS (Fig. 3B). These results are consistent with the EMSA results above, demonstrating that SA and PAS are ligands of Rv2887 *in vivo*, and with previous reports suggesting that ligands of MarR family proteins are often phenolic compounds^1, 3, 4^.

### Structure of the Rv2887-DNA complex reveals an indirect mechanism of DNA sequence readout

**Figure 4:**
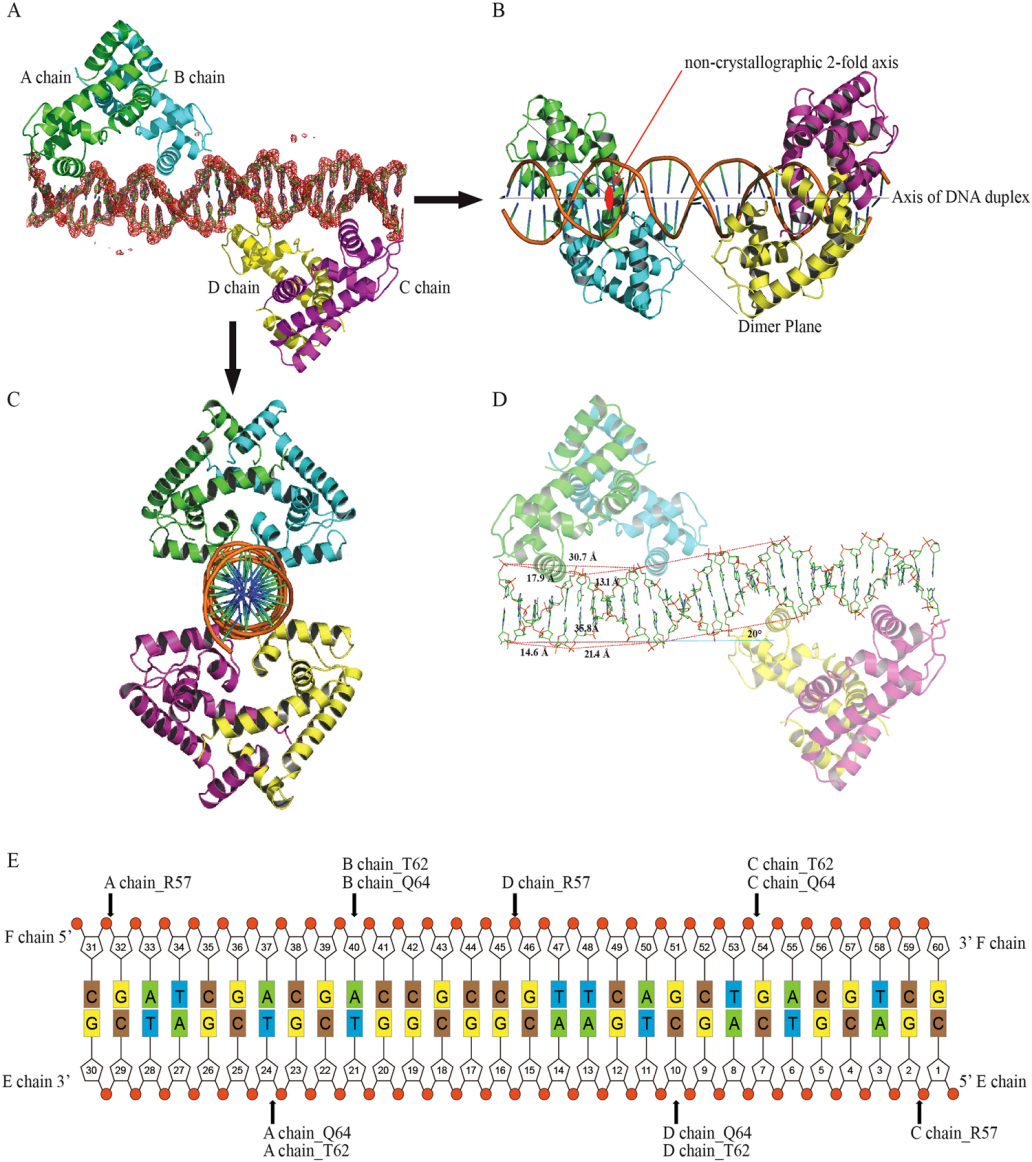
The crystal structure of the Rv2887-DNA complex. (**A**,**B**,**C**) Orthographic views of the complex structure of a 30 bp DNA and two Rv2887 dimers in a unit cell. The DNA is surrounded by its ligand-omit 1FO-FC electron density map (red), contoured at 3.0 σ. The non-crystallographic 2-fold axis of the Rv2887 dimer is shown as a red ellipse and is perpendicular to the axis of the DNA duplex. The angle between the axis of the DNA duplex and the triangle-like shape of the protein dimer is ~40°. (**D**) The lengths of the major groove, the minor grove and the DNA duplex helix in one flank of the bound DNA are different from those in the other flank, and the angle of distortion is ~20°. (**E**) Schematic representation of the Rv2887-DNA interaction. Hydrogen bonds are indicated by arrows from the residues to the nucleotides.

To investigate the DNA binding mode of Rv2887, Rv2887 was crystallized in the presence of DNA duplexes of different lengths derived from a sequence upstream of the Rv0560c transcriptional start site containing the 27-bp binding site identified by DNase I footprinting of the Rv0560c promoter region (Fig. 1B). The best crystals were formed when a 30 bp duplex was used and these were used to determine the complex structure at a resolution of 2.5 Å (Supplementary Table S1). The electron density map obtained was of high-enough quality to unambiguously build the DNA model. The structure showed that two Rv2887 dimers (dimer AB consisting of chains A and B, and dimer CD consisting of chains C and D) bound to the 30-bp DNA duplex (chains E and F) (Fig. 4A). Each protein dimer exists as a triangular shape, similar to that of the *apo*, SA-bound and PAS-bound structures, and has a Cα atom r.m.s.d. relative to the *apo*, SA-bound and PAS-bound structures of 2.0, 1.6, 1.5 Å, respectively. Each dimer sits independently over one flank of the DNA, covering a 15 bp stretch of DNA (Fig. 4A). The angle between the triangular protein dimer and the DNA double helix is about 40° (Fig. 4B). The two Rv2887 dimers are positioned on nearly opposite faces of the DNA, with symmetry like a 2_1_ screw axis (Fig. 4C). The two protein dimers simultaneously bind to the 30-bp DNA in a non-crystallographic 2-fold symmetrical manner (Fig. 4A–C), similar to that previously reported for the QacR-DNA complex and the ST1710-DNA complex structure^14, 29^. Rv2887 interacts with DNA mainly via insertion of helix α4 into the major groove of the DNA where it forms Van der Waal and hydrogen bonds (Fig. 4D). The four protomers observed in the complex structure form similar hydrogen bonds with DNA (Fig. 4D and E). The side chain of Arg57 in chains A, C, and D forms hydrogen bonds with the phosphate groups of DNA bases F31, E1 and F46, respectively. However, the side chain of Arg57 in chain B was not observed to form a hydrogen bond with DNA. The side chains of residues Thr62 and Gln64 in chains A and D form hydrogen bonds with the phosphate groups of the E25 and E10 guanine nucleotides of DNA chain E, respectively, and the side chains of residues Thr62 and Gln64 in chains B and C form hydrogen bonds with the phosphate groups of the F40 and F55 thymine nucleotides, respectively (Fig. 4E). Protein sequence alignment indicates that residue Arg89 of Rv2887, located at the winged loop, along with Arg81, which Warrior *et al*. demonstrated to be important for the binding of Rv2887 to Rv560c promoter DNA^20^, are conserved among members of the MarR family of proteins (Supplementary Fig. S1) and their corresponding residues in regulator ST1710 play a major role in protein-DNA interactions^14^. However, Rv2887 interacts with DNA mainly via insertion of helix α4 into the major groove of the DNA rather than via the winged loop region. This can easily be seen when comparing the Rv2887-DNA complex with the ST1710-DNA complex; the distance of the protein from DNA in Rv2887 is shorter than that in the ST1710-DNA complex (Supplementary Fig. S2).

We next investigated the mechanism by which Rv2887 recognizes DNA sequences. The length of a full turn in the DNA helix in the flank where Rv2887 dimer AB binds is 30.7 Å (the distance between the phosphate atoms of F31 and F41, Fig. 4D), while the length of the major groove in the flank where the Rv2887 dimer binds by insertion of helix α4 is 17.9 Å (the distance between the phosphate atoms of F31 and E25), and that of the minor groove is 13.1 Å (the distance between the phosphate atoms in E25 to F41). By contrast, these three distances on the complementary strand were measured as 35.8 Å (E20 to E30), 21.4 Å (E20 to F36), and 14.6 Å (F36 to E30), respectively (Fig. 4D). In an ideal B-form DNA duplex modelled by COOT^30^, these distances are 33.8 Å, 20.6 Å, and 13.2 Å, respectively. The length of the major groove (17.9 Å) that interacts with the protein is significantly shorter in the complementary strand that does not interact with the protein and in the ideal DNA duplex model (21.4 and 20.6 Å, respectively). Binding of the protein to the DNA thus appears to pull on the two termini of the major groove, making the major groove more compact and bending the DNA duplex towards the protein by an angle of 20° (Fig. 4D). This bending of the DNA duplex may destroy hydrogen bonds between DNA base pairs that would otherwise form. Interestingly, the DNA sequence CGATCG (E25-E30 and F31-36) described above that is bent by Chains A and B (Fig. 4D) is palindromic, while the sequence bent by Chains C and D is not palindromic. However, both these DNA sequences recognized by Rv2887 have a high G/C content. We propose that Rv2887 recognizes a specific DNA sequence via an indirect readout mechanism^31^ and that a high G/C content in the DNA sequence is required to stabilize the bend in the DNA and ensure the major groove is of a specific length suitable for protein binding. An indirect readout mechanism, such as that postulated for *Staphylococcus aureus* MarR family protein MepR^31^, may explain how Rv2887 can act as a global regulator, binding different DNA sequences and thereby regulating the expression of many genes.

### SA and PAS stabilize a conformation of Rv2887 incompatible with DNA binding

**Figure 5:**
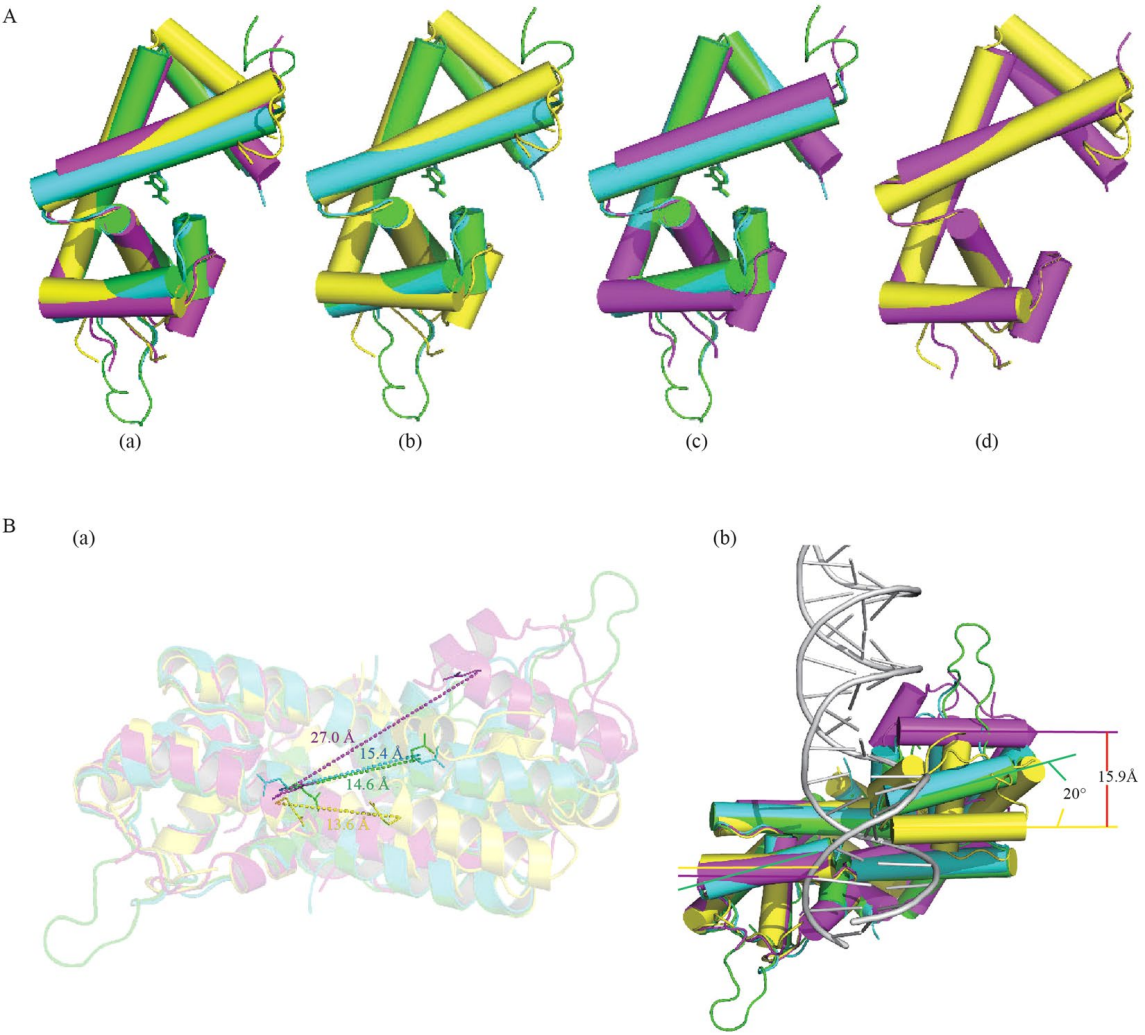
Ligand binding induces significant conformational change in Rv2887. (**A**) (a) Monomeric structure superposition of apo (yellow), SA- (green), PAS- (blue) and DNA-bound (purple) Rv2887. (b) The binding of ligand SA/PAS induces the DNA binding domain (wHTH) to rotate up towards the dimerization interface by nearly 15~20°. (c) No significant conformational change was observed in the Rv2887 protomer upon ligand or DNA binding, except for the rotation of the wHTH domain. (d) The main difference between the apo and DNA-bound forms of Rv2887 is in the N-terminal. (**B**). Dimeric structure superposition of apo (yellow), SA- (green), PAS- (blue) and DNA-bound (purple) Rv2887. (a) Major differences between different dimers include different distances between the two DNA recognition helices (i.e. the distance between the Q64 and Q64′ Cα atoms) in different protein dimers (apo-: 13.6 Å; SA-:14.6 Å; PAS-:15.4 Å; DNA-: 27.0 Å). (b) Different orientations of the α4 helices in the ligand- or DNA-bound complexes result in different distortions of the dimer.

Superposition of the monomer of apo-Rv2887, the Rv2887-ligand complexes and the Rv2887-DNA complex reveals that conformational changes take place in the N-terminal and the winged-HTH (wHTH) domain on ligand or DNA binding (Fig. 5A-a). Specifically, binding of SA or PAS causes the DNA binding (wHTH) domain to rotate up towards the dimerization interface by 15~20°, and the N-terminal to rotate forward to the ligand binding pocket. Rotation of the wHTH domain of one monomer and the N-terminal region of the other Rv2887 monomer result in the formation of a buried binding pocket for ligands, over which the N-terminal residues form a ‘lid’ (Fig. 5A-b). The main conformational change in the Rv2887 monomer between its apo- and DNA-bound forms is a forward rotation of the N-terminal, while the main change between the DNA- and ligand-bound forms is rotation of the wHTH domain. Rotation of the N-terminal plays a key role in the dimerization of Rv2887 (Fig. 5A-d).

Superposition of the apo-Rv2887, Rv2887-ligand and Rv2887-DNA complexes through the monomer of Rv2887 reveals that the main differences between these states are the distance between the two α4 DNA recognition helices in the Rv2887 dimer and the rotation of the monomers within the dimer. The distance between the two DNA recognition helices in the dimer, i.e. the distance of the two major grooves of DNA bound by the Rv2887 dimer, determines the length of DNA that can be sequestered by the two recognition helices, and is measured using the distance between the Gln64 Cα and Gln64′ Cα atoms. This distance, 27.0 Å, is equivalent to 7 bp DNA, the length of the sequence which is clamped by the wHTH domains (Figs 4D and 5B). The distances between the Gln64 Cα and Gln64′ Cα atoms are 13.6 Å, 14.6 Å and 15.4 Å in the *apo*, SA-bound and PAS-bound forms, respectively (Fig. 5B). The distance in the Rv2887-DNA complex is longer than that in the ligand-bound complexes, indicating that the conformational changes in the Rv2887-DNA complex may favor insertion of helix α4 into the major groove of the DNA, whereas the conformational changes in the ligand-bound Rv2887 complexes are incompatible with DNA binding.

Conformational changes in the wHTH domain are a common mechanism of gene repression in the MarR family; however, the detailed conformational changes induced by ligands or DNA are different in each case^14–16, 31, 32^. In an attempt to identify the unique characteristics of the ligand-regulated DNA binding mechanism of Rv2887 in *M. tuberculosis*, we compared our Rv2887-related structures with those of ST1710^14, 27^, OhrR^32, 33^ and SlyA^16, 34^ (Supplementary Fig. S2). In contrast to Rv2887, SA binding does not induce any significant conformational changes in ST1710 and the distance between the wHTH domain in the dimer is only reduced by ~10 Å in the ST1710-DNA complex compared to its *apo* and SA-bound states^14, 27^. The wHTH motif of OhrR binds DNA on insertion of the α4 recognition helix more deeply into the major groove than Rv2887, leading to much greater widening and deepening of the groove, however, unlike Rv2887, there is little difference in the distances between the α4 recognition helices of *apo* OhrR and the OhrR-DNA complex. In SlyA, SA binding causes a large motion in the α4 helices of both subunits, but the SA-bound SlyA complex shows few large conformational changes in its DNA-bound complex. In summary, the distance between the wHTH domain in the dimer in the SA- or PAS-bound Rv2887 complex is greater than in that in ST1710, OhrR and SlyA, and SA/PAS-binding also induces translocation of the wHTH domains in Rv2887 by ~30° towards the 2-fold axis. The conformational changes observed in these MarR family protein complexes suggest that while their mechanisms share common features, the details vary considerably from protein to protein in accordance with their cognate DNA and ligand molecules.

## Discussion

Here, using a structural approach, we have elucidated the DNA-binding mechanism of *M. tuberculosis* protein Rv2887. Rv2887 functions as a transcriptional repressor; binding of effector molecule SA, or its structural analogue PAS, to the binding pocket of Rv2887 induces conformational change in its DNA binding domain, shrinking the distance between the α4 helices and preventing binding of Rv2887 to the promoter region of Rv0560c, triggering the expression of this SAM-dependent methyltransferase.

Our crystal structures confirm that *M. tuberculosis* Rv2887 is a typical MarR family protein. It is a dimer with a triangular shape consisting of six α-helices and two β-strands that form a dimerization domain (helices α1, α5, and α6), and a wHTH DNA-binding domain (helices α3 and α4 separated by a wing motif comprising two antiparallel β-strands and their connecting loop) (Fig. 2B). Rv2887 binds two SA or PAS molecules per dimer. Binding occurs in a similar manner (Fig. 2D–F) and results in large conformational changes within Rv2887 that attenuate its binding to DNA (Figs 4 and 5). The DNA binding ability of MarR family proteins is facilitated by their conformational flexibility. We found that the distances between the α4 helix of the wHTH domains in the dimer of the Rv2887-DNA complex are larger than in its *apo* and ligand-bound forms and that these conformational changes are required to facilitate insertion of the recognition helix α4 of the wHTH motif into the major groove of DNA during DNA binding (Fig. 4). We observed differences between the Rv2887-DNA structure and other reported MarR-DNA complexes that may lead to variation in DNA-binding characteristics. Superposition of the Rv2887-DNA structure and the *B. subtilis* OhrR-DNA complex structure^32^, for example, showed that the DNA duplex is about 7 Å further away in the Rv2887-DNA structure than in the OhrR-DNA structure. The α4 helix of Rv2887 fits more easily into the major groove of DNA than that of OhrR, and this difference is confirmed by the fewer hydrogen bonds observed in the Rv2887-DNA structure than in the OhrR-DNA structure^32^. Notably, binding of Rv2887 to DNA induces a large conformational change in the α4 helices of both protomers, resulting in the distance between the two α4 helices in the Rv2887 protein dimer being significantly greater than in the *B. subtilis* OhrR-, *S. enterica* SlyA- and *S. tokodaii* ST1710-DNA complexes^14, 16, 32^. In addition, superposition of Rv2887 and SlyA or OhrR reveals that the N-terminus of the Rv2887 α4 helix (residues 62–64), rather than the middle part of this helix, as in the other MarR family proteins, is clamped by the major groove.

The mechanism of DNA binding attenuation in MarR family proteins is related to conformational changes in the wHTH domain^1–3^. Here, the beta wing in the DNA-bound form of Rv2887 was found to be disordered, but could be traced clearly in the two ligand-bound forms. The beta-wing in the SlyA-DNA and ST1710-DNA complex structures has previously been reported to have an ordered secondary structure that is stabilized by DNA binding^14, 16^. This difference may be due to the rich proline, serine and glycine content of the beta wing of Rv2887 (82–92, PASVSSGRSLP). Rv2887 appears to use an indirect readout mechanism for recognizing specific DNA sequences. Examination of the Rv2887-DNA complex structure indicates that Rv2887 does not interact directly with the base moiety of DNA, suggesting it does not directly recognize a specific DNA sequence via hydrogen bonding between the protein and DNA sequence. A similar observation was noted in a study on the structure of MarR protein SlyA in complex with DNA in which there was only one bidentate hydrogen bond between Arg65 and guanine14^16^. The authors suggested that SlyA recognizes DNA fragments irrespective of sequence heterogeneity and proposed that SlyA, a global regulator, can readout DNA sequences in an indirect manner and thus control the expression of many genes. Given that Rv2887 also seems to function as a global regulator, we propose that Rv2887 may also recognize DNA sequences with which it has high affinity in an indirect manner.

The involvement of MarR family proteins in the mar phenotype in *M. tuberculosis* appears to be complex. The mar phenotype, induced by exogenous SA in both Gram negative and Gram positive bacteria, including *M. tuberculosis*, is thought to involve the induction of efflux pumps via alleviation of transcriptional repression by MarR family proteins^3, 4^. The mechanism of this SA-induced mar phenotype in *M. tuberculosis*, however, is poorly understood as none of the genes whose expression is induced by SA exposure are known efflux pumps^35^. The expression of Rv0560c is upregulated 30-fold in the presence of SA, and its structural analogue antituberculosis drug PAS also induces the expression of Rv0560c^23^. Rv0560c was recently confirmed to be a SAM-dependent methyltransferase^36^; it was shown to *N*-methylate 14, a compound with antitubercular activity, abolishing its activity. It will be interesting to determine if Rv0560c also *N*-methylates PAS and if *N*-methylated PAS also loses its antibacterial activity. Further investigation to elucidate the full physiological role of Rv0560c and its regulation in *M. tuberculosis* is necessary. The regulatory mechanism of Rv0560c may be complex and involve more than one transcriptional regulator; not all compounds that induce the expression of Rv0560c, for example fenofibrate, do so via MarR family protein Rv2887; fenofibrate does not interact with Rv2887, but can induce high levels of Rv0560c expression^23^. In addition, mutations in the upstream region of a putative alternative start site also affect the expression of Rv0560c^23^, suggesting there may be more than one repressor protein involved in Rv0560c regulation.

To conclude, we have identified ligands of *M. tuberculosis* MarR family protein Rv2887 and its DNA binding sequence and have elucidated its regulatory mechanism based on structures of Rv2887 in its *apo-* form and in complex with SA, PAS and DNA. The structures presented enrich our understanding of the DNA-binding mechanism of the MarR family of proteins. Structural information on additional ligand-bound MarR family protein complexes and DNA-bound MarR regulators will be required to provide a more generalized description of binding pocket properties in MarR proteins as well as to understand more clearly how they change their conformation in the presence of various ligands to mediate protein-DNA interactions. Given the important role played by MarR family proteins in the mar phenotype of bacteria, our study of the mechanism and regulatory pathway of MarR protein Rv2887 in *M. tuberculosis* provides insight which may lead to greater understanding of drug resistance mechanisms in *M. tuberculosis*. Further functional and structural studies on MarR family proteins are needed to facilitate the design and development of drugs that specifically target MarR-DNA interactions.

## Materials and Methods

### Overexpression and purification of Rv2887

The Rv2887 gene was amplified from purified genomic DNA of *M. tuberculosis* H37Rv using the following PCR primers: GGAATTCCATATGATGGGTCTAGCCGATGAC (forward) and CCCAAGCTT CTAGTCGGACCCGAGCTTC (reverse), and ligated into the vector pET-28a(+) (Novagen), generating plasmids encoding Rv2887 protein with a 6× His tag at its N-terminus. Plasmid pET28aΩRv2887 was then transformed into *E. coli* BL21(DE3) cells. Cultures were grown to an OD_600_ between 0.6 and 0.8, and Rv2887 expression was induced upon addition of IPTG at a final concentration of 0.5 mM and allowed to proceed for 16 h at 16 °C. Bacterial cells were then suspended in ice-cold lysis buffer containing 20 mM Tris (pH 7.4), 10 mM imidazole, and 500 mM NaCl, and subjected to high-pressure homogenization. After removing cell debris by centrifugation for 40 min at 15,000 rpm (4 °C), the crude lysate was loaded onto a Ni^2+^ -chelating column pre-equilibrated with 20 mM Tris buffer (pH 7.4) containing 10 mM imidazole and 500 mM NaCl. After washing with six column volumes of buffer containing 80 mM imidazole, 500 mM NaCl and 20 mM Tris (pH 7.4), Rv2887 protein was eluted with two column volumes of buffer containing 300 mM imidazole, 500 mM NaCl and 20 mM Tris (pH 7.4), and further purified by Superdex 200 (GE Healthcare) size-exclusion chromatography. The final purified protein was concentrated by centrifugation and stored in 20 mM Tris buffer (pH 7.4) containing 150 mM NaCl, and 5% glycerol at −80 °C.

Selenomethionine (Se-Met)-labeled Rv2887 was produced by inhibiting endogenous methionine biosynthesis in M9 minimal media supplemented with specific amino acids and Se-Met and then purified as described above. Single amino acid substitutions in Rv2887 were accomplished using the method of Shenoy *et al*.^37^. Mutants were expressed and purified using the procedure described above for WT Rv2887, and were stored in the same buffer.

### Electrophoretic mobility shift assays

DNA probes were amplified by PCR using KOD polymerase and primers biotin-0560-probe-F: GCCCATCACGATGCGCTCCACCGC and 0560-probe-R: CGAACTCAAGATCCAGCGATTCAG. Labeled PCR products were purified using an Omega Cycle Pure Kit. DNA concentrations were determined using a Nanodrop ND-1000 spectrophotometer. The biotin-labeled DNA fragments were incubated with purified Rv2887 protein in EMSA binding buffer (Beyotime, China) which includes poly(dI-dC) to eliminate nonspecific binding between protein and DNA. A labeled random DNA of the some length as the target probe (generated using the web site http://www.geneinfinity.org/sms/sms_dnarandom.html) was used as a control. Each reaction contained 1 nM PCR-amplified probes and varying concentrations of protein Rv2887. The effect of ligands SA and PAS on the binding of Rv2887 to DNA was determined by adding varying concentrations of ligands to the Rv2887-DNA reaction mixture. Reaction mixtures were incubated at 25 °C for 30 min, loaded onto a 5% PAGE gel and run in 0.5× TBE buffer at 120 V for 50 min on ice. DNA mobility shifts were subsequently detected using a Chemiluminescent EMSA Kit (Beyotime, GS009).

### DNase I footprinting assays

Templates for DNase I footprinting were amplified by PCR using a FAM-labeled primer pair (6-FAM-0560-probe-F: GCCCATCACGATGCGCTCCACCGC and 0560-probe-R: CGAACTCAAGATCCAGCGATTCAG). DNase I footprinting assays were performed according to the method of Wang *et al*.^38^. For each assay, probes (200 g) were incubated with different amounts of Rv2887 in a total volume of 40 µl EMSA binding buffer (Beyotime, China). After incubation for 30 min at 25 °C, 10 µl solution containing about 0.015 units DNase I (Promega) and 100 nM freshly prepared CaCl_2_ was added and further incubated for 1 min at 25 °C. The reaction was stopped by adding 140 µl DNase I stop solution (200 mM unbuffered sodium acetate, 30 mM EDTA and 0.15% SDS). Samples were then extracted with phenol/chloroform, precipitated with ethanol and pellets were dissolved in 30 µl ddH_2_O water. Preparation of the DNA ladder and electrophoresis conditions were the same as described by Wang *et al*.^38^, except that a GeneScan-LIZ500 size standard (Applied Biosystems) was used.

### Isothermal titration calorimetry

Binding of SA, PAS and gemfibrozil to Rv2887 was observed using an ITC200 (isothermal titration calorimeter 200 μl cell; Microcal, Northampton, MA, USA) at room temperature. Protein Rv2887 and ligand samples were dissolved in the same buffer (20 mM Tris-HCl, pH 7.4, 150 mM NaCl) and were degassed before loading into the cell and syringe. Binding experiments were carried out with the Rv2887 protein solution in the cell and the ligand solution as the injectant. Ligand solutions were injected into the Rv2887 protein solution in 2 μl aliquots at 120 s intervals, with stirring at 1000 rpm. The association constant, Ka, and the number of binding sites, n, were calculated and analyzed with MicroCal Origin 5.0 software.

### RNA isolation and quantitative real-time PCR

Cultures of *M. tuberculosis* strain H37Rv grown to log phase were divided into 50 ml aliquots. SA and PAS were added to a final concentration of 2 mM before incubating for 90 min at 37 °C. Untreated cultures were used as controls. Total RNA was extracted from culture pellets using a FastRNA Pro Blue kit (MP Bio) according to the manufacturer’s guidelines. Following extraction, RNA was treated with DNase I to degrade all DNA present. RT-PCR was carried out with a TransScript II Green Two-Step qRT-PCR SuperMix kit (TransGen, China) according to the manufacturer’s instructions. Synthesis of complementary DNA (cDNA): mixtures containing 4 μl 5× TransScript II All-in-One SuperMix for qPCR, 2 μg total RNA, 1 μl gDNA Remover and RNase-free water (up to a total volume of 20 μl) were incubated at 50 °C for 15 min. qRT-PCR was performed on a Bio-Rad CFX96 real-time system, each reaction having a final volume of 20 μl and comprising 10 μl 2× TransStart Top Green qPCR SuperMix, 2 μl of the cDNA, 0.4 μl of each primer (10 μM), and DEPC water. Reaction conditions: one cycle of 94 °C for 30 s, 40 cycles of 94 °C for 5 s and 60 °C for 30 s. The fold change in the target gene relative to housekeeping gene *sigA* was calculated as: Fold change = 2^−^Δ(ΔCT), where ΔC_T_ = C_T(target)_ − C_T(sigA)_, and Δ(ΔCT) = ΔC_T(Treated)_ − ΔC_T(control)_. Primers used for qRT-PCR analysis were: Rv0560c-F: TCGGCATGAAGCAGCGAAGCG; Rv0560c-R: TCGACCGGCATGGAGTGGAACAGC; SigA-F: TGCGGCTACGCTTCGGCCTTAC; and SigA-R: TGGCGCAACTTCGACATAGTCTTGGA.

### Rv2887 cystallization

#### *apo* Rv2887

Purified Rv2887 protein (15 mg/ml) in 20 mM Tris buffer (pH 7.4) containing 150 mM NaCl and 5% glycerol was used in all crystallization experiments. Cystallization experiments were performed at 16 °C using the hanging-drop vapor-diffusion method. Hampton Crystal Screen Kits (Index, Natrix, Crystal Screen I and II) were used to determine initial crystallization conditions. Typically, a 1 μl drop of protein solution was mixed with an equal volume of screening solution and equilibrated over a reservoir containing 0.2 ml of reservoir solution. The best crystals were obtained in a final reservoir solution containing 0.1 M citric acid (pH 3.5) and 2.0 M ammonium sulfate.

For phasing experiments, crystals were exchanged into drops of Hg^2+^ using a Hampton Research Heavy Atom Screen kit and allowed to soak overnight.

Cryoprotection was achieved by raising the glycerol concentration stepwise to 20% in 5% increments.

### Rv2887-Ligand complex

To obtain Rv2887-ligand complex crystals, a solution containing Rv2887 (15 mg/ml) and a 5-fold molar excess of SA or PAS (SA was solubilized in buffer containing 20 mM Tris (pH 7.4), 150 mM NaCl, and 5% glycerol, and PAS in DMSO) was incubated at 25 °C for 45 min, centrifuged briefly and used immediately for crystallization using the same procedures as for *apo* Rv2887.

### Rv2887-DNA complex

Protein Rv2887 was concentrated to 30 mg ml^−1^ in crystallization buffer (20 mM Tris buffer (pH 7.4) containing 150 mM NaCl and 5% glycerol). Complementary pairs of DNA oligonucleotides of different lengths (27–32 bp) and ends (blunt or sticky ends), designed according to DNase I footprinting assay results, were obtained from Sangon Biotech and DNA duplexes were reconstituted by annealing oligonucleotide pairs overnight in crystallization buffer. Rv2887 and annealed oligonucleotides were mixed together in a ratio of 1 Rv2887 dimer: 1.2 double-stranded oligonucleotides and incubated at 25 °C for 2 h. Cystallization experiments were performed as above using Hampton Crystal Screen Kits (Index, Natrix, Crystal Screen I and II). The best Rv2887-DNA crystals were obtained with a solution of 0.1 M Potassium chloride, 0.025 M Magnesium chloride hexahydrate, 0.05 M Sodium cacodylate trihydrate pH 6.0, and 15% V/V 2-Propanol, and a blunt-end 30 bp-mer DNA. Crystals appeared in 3–6 days. Se-Met-labeled Rv2887-DNA crystals were obtained under the same conditions. Rv2887-30-mer crystals were cryoprotected by a two-step transfer process in which glycerol was added to the drop to a final concentration of 10%.

### Data collection and structure refinement

The *apo* Rv2887 protein dataset was collected on a Rigaku R-Axis IV++ image plate using Cu Kα radiation (λ = 1.5418 Å) at 100 K. Protein-ligand complex datasets were collected on beamline BL17U at the Shanghai Synchrotron Radiation Facility (Shanghai, China). All datasets were processed with iMOSFLM and scaled with SCALA (CCP4 program suite)^39–42^. Phase determination of Rv2887 was performed with PHASER using a Hg^2+^-derivative crystal obtained from the above screen performed with a Hampton Research Heavy Atom Screen kit, and the model was built manually with COOT^30^. Model refinement was carried out with PHENIX^43^. The structure of the Rv2887 protein-ligand complex was solved by molecular replacement with PHASER using the Rv2887 structure as a search model. Model building and structural refinement were performed with COOT and PHENIX, respectively. As the Rv2887-DNA complex could not be phased successfully using the molecular replacement method, we collected and processed Se-SAD data using Se-Met labeled Rv2887. Data collection and refinement statistics are summarized in Supplementary Table S1. All structural figures were rendered in PyMOL (http://www.pymol.org).

### Protein Data Bank accession codes

The atomic coordinates and structure factors for the *apo* Rv2887, Rv2887-SA complex, Rv2887-PAS complex, and Rv2887-DNA complex have been deposited in the PDB with accession numbers 5HSM, 5X80, 5X7Z, and 5HSO, respectively.

## Electronic supplementary material

Supplementary Information
