# Supplementary material for: Structural analysis of the regulatory mechanism of MarR protein Rv2887 in M. tuberculosis
Source: Sci Rep. 2017 Jul 25;7:6471. doi: 10.1038/s41598-017-01705-4 (PMC5526998; doi:10.1038/s41598-017-01705-4)
Supplement: Supplementary file 1 — Supplementary Information [file 41598_2017_1705_MOESM1_ESM.pdf]

Supplementary Information

Structural analysis of the regulatory mechanism of MarR protein Rv2887 in *M. tuberculosis*

Yun-Rong Gao[1,2,8,†], De-Feng Li[2,†], Joy Fleming[1,2,†], Ya-feng Zhou[1], Liu Ying[3], Jiao-Yu Deng[4], Lin Zhou[5], Jie Zhou[6], Guo-Feng Zhu[2], Xian-En Zhang[2], Da-Cheng Wang[2,*], Li-Jun Bi[1,2,7,*]

1. School of Stomatology and Medicine, Foshan University, Foshan 528000, Guangdong Province, China
2. Key Laboratory of RNA Biology & National Laboratory of Biomacromolecules, Institute of Biophysics, Chinese Academy of Sciences, Beijing 100101, China
3. Shanghai Key Laboratory of New Drug Design, School of Pharmacy, East China University of Science and Technology, Shanghai 200237, China
4. State Key Laboratory of Virology, Wuhan Institute of Virology, Chinese Academy of Sciences, Wuhan 430071, China
5. Center for Tuberculosis Control of Guangdong Province, Guangzhou 510630, China
6. The 4th Peoples' Hospital, Foshan 528000, Guangdong Province, China
7. Guangdong Province Key Laboratory of TB Systems Biology and Translational Medicine, Foshan 528000, China
8. University of the Chinese Academy of Sciences, Beijing 100049, China

[†] These authors contributed equally to the paper as first authors.

[*] To whom correspondence should be addressed: Tel: +86-10-64888464, Fax: +86-10-64871293, E-mail: blj@ibp.ac.cn

Correspondence may also be addressed to Da-Cheng Wang: Tel: 86-10-64888547, Fax: +86-10-64888560, E-mail: dcwang@ibp.ac.cn

Supplementary Table S1. Crystallographic data collection and refinement statistics

Crystals	*apo* Rv2887	Rv2887-SA	Rv2887-PAS	Rv2887-DNA
Data collection				
Space group	C2221	P21	C2221	P1
Cell dimensions				
a, b, c, (Å)	53.54, 71.29, 72.88	57.97, 44.15, 122.16	86.81, 86.87, 43.56	48.22, 48.23, 101.11
α, β, γ, (°)	90, 90, 90	90, 90.42, 90	90, 90, 90	89.95, 90.04, 107.13
Wavelength (Å)	1.5418	1.5418	0.9791	0.9795
Resolution (Å)[a]	36.44-1.90 (2.00-1.90)	57.97-2.40 (2.53-2.40)	27.45-2.20 (2.32-2.20)	36.21-2.50 (2.56-2.50)
R_{merge} (%)[b]	4.7 (30.5)	6.1 (20.7)	11.7 (82.1)	4.0 (63.0)
$<I/\sigma(I)>$	21.3 (5.2)	11.5 (5.0)	11.0 (3.2)	15.4 (2.0)
Completeness (%)	100 (100)	89.8 (82.8)	99.7 (100)	99.5 (99.8)
Redundancy	6.9 (6.9)	3.7 (3.7)	6.7 (6.8)	3.4 (3.5)
Wilson plot B	30.9	42.0	40.8	63.2
Refinement				
No. reflections	11333 (1632)	21945 (2932)	8664 (1245)	29580 (2233)
R_{work}/R_{free}^{+}(%)	18.4/20.4	23.1/25.9	20.5/24.8	24.0/27.8
R.m.s.d.				
Bond lengths (Å)	0.007	0.007	0.009	0.004
Bond angles (°)	1.121	1.257	0.995	0.924
No. of non-H atoms	1067	4045	1014	4896
Protein/DNA	976	3851	969	4896
Ligand/ion	10	50	11	-
Water	81	144	34	-
Average *B* factors ($Å^2$)	40.5	42.0	57.7	89.0
Protein/DNA	39.5	42.1	57.8	89.0
Ligand/ion	94.6	40.6	62.8	-
Water	45.4	40.0	52.1	-
Ramachandran plot, Residues in (%)				
Favored	100	98.81	95.38	98.95
Allowed	0	1.19	2.31	1.05
Outliers	0	0	2.31	0

[a]Data for the highest resolution shell is shown in parentheses.

[b]$R_{merge}=\Sigma_{hkl}\Sigma_i|I_i(hkl)-<I(hkl)>| / \Sigma_{hkl}\Sigma_i I_i(hkl)$ where $I_i(hkl)$ is the *i*th observation of a symmetry equivalent reflection hkl.

Supplementary Table S2. Association constants of SA and Rv2887 mutants

**Protein Rv2887**	**Ligand**	**Ka (M)**
WT Rv2887	SA	$2.45E4 \pm 1.23E3\ M^{-1}$
Rv2887_R42A	SA	$2.51E3 \pm 131\ M^{-1}$
Rv2887_D114A	SA	$4.65E3 \pm 389\ M^{-1}$

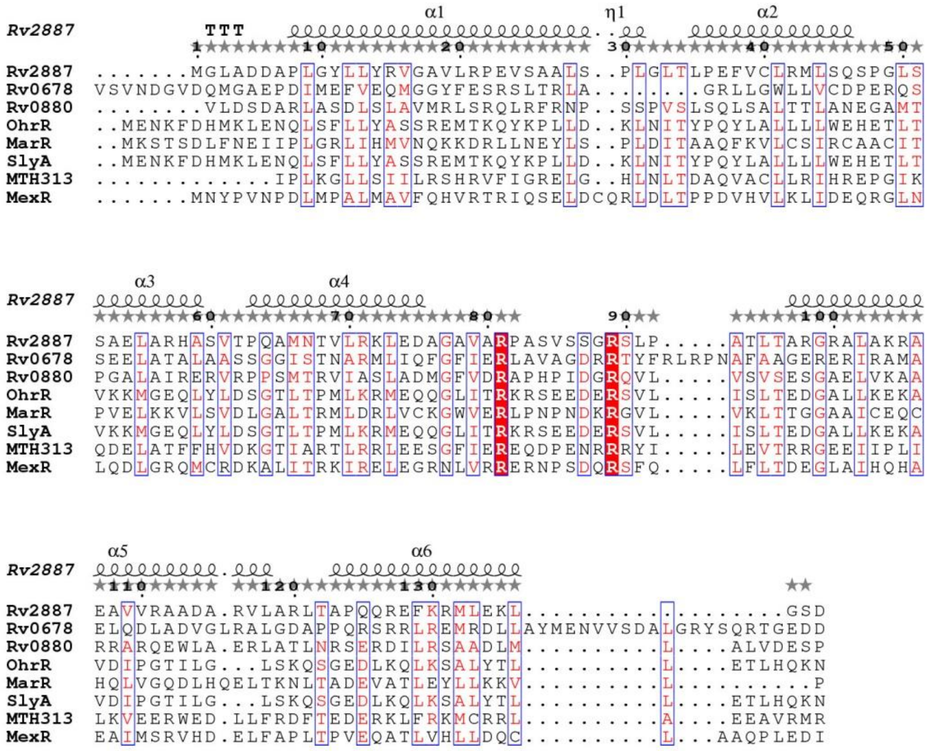

Supplementary Figure S1. Sequence alignment of Rv2887 and its homologous proteins from *M. tuberculosis* and other species (*M. tuberculosis* Rv2887, Rv0678 and Rv0880, *B. subtilis* OhrR, *E.coli* MarR, *S.enterica* SlyA, *M.thermoautotrophicum* MTH313, *P. aeruginosa* MexR).

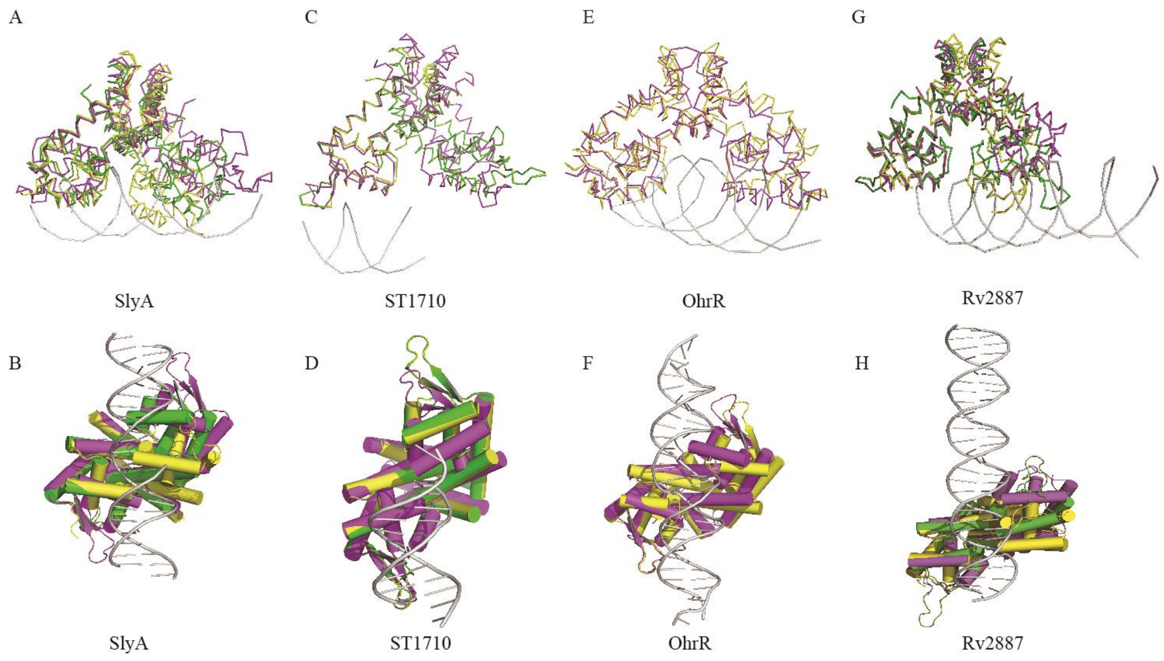

Supplementary Figure S2. Dimeric structure superposition of apo (yellow), ligand-(green) and DNA-bound (purple) MarR family proteins SlyA, ST1710, OhrR, and Rv2887. Upper figures (A, C, E, G) are ribbon forms, lower figures (B, D, F, H) are cartoon forms with cylindrical helices.
